# Repair of Bile Duct Injury Using Indocyanine Green Following Laparoscopic Cholecystectomy

**DOI:** 10.7759/cureus.49312

**Published:** 2023-11-23

**Authors:** Satoshi Tokuda, Akitsugu Fujita, Akihiko Takagi, Hideyuki Kanemoto, Noriyuki Oba

**Affiliations:** 1 Department of Gastroenterological Surgery, Shizuoka General Hospital, Shizuoka, JPN

**Keywords:** jaundice, cholecystitis, indocyanine green, cholecystectomy, bile duct injury

## Abstract

Bile duct injury (BDI) is a potential complication that may arise during cholecystectomy and continues to occur with a certain frequency in the present day. Numerous reports have been published regarding the utilization of indocyanine green (ICG) for the prevention of biliary injury, and we feel that the importance of ICG is being recognized. In this context, we present a case wherein a BDI occurred following cholecystectomy, and ICG was employed for the safe repair of the biliary tract.

## Introduction

Bile duct injury (BDI) is a potential complication of cholecystectomy, with reported incidence rates of 0.1-0.2% for open and 0.4-0.6% for laparoscopic procedures [1]. Recently, robotic surgery has also been introduced, but BDI incidence has also been observed in this [2]. BDI additionally exerts influence on the prognosis, as evidenced by a documented mortality rate of 3.5% [3]. The range of patients receiving an intraoperative diagnosis of BDI fluctuates between 25% and 92%, contingent upon the particular report; however, it is not unusual for BDI confirmation to occur in the postoperative phase [4]. The usefulness of using indocyanine green (ICG) to reduce the risk of BDI during initial cholecystectomy has been reported [4]; however, this technique is limited to the prevention of BDI. Here, we report a case in which intraoperative ICG was used for the successful repair of a bile duct injury that occurred after open cholecystectomy at a different hospital. This case is a report of repair using ICG after a long time-lapse from the onset of BDI. There are few similar reports, and we consider it a significant paper.

## Case presentation

**Figure 1:**
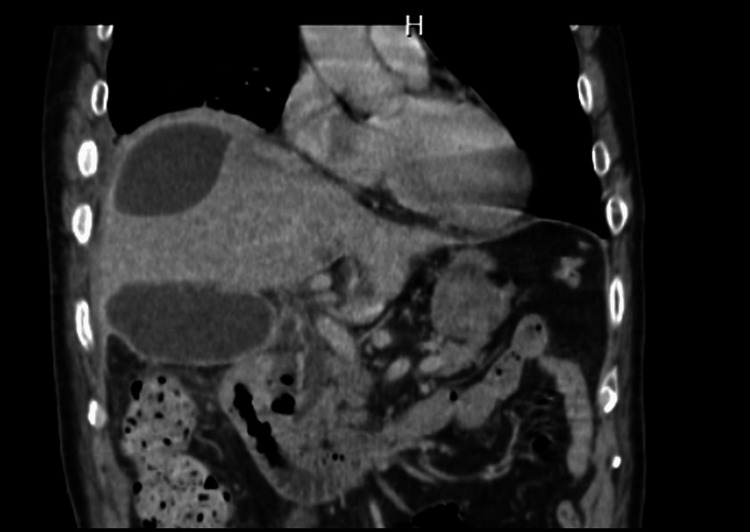
CT before initial surgery The patient had an enlarged gallbladder, wall thickening, and an abscess formation under the right diaphragm.

A 76-year-old Japanese male presented to his previous physician with complaints of pain in his right hypochondrium. Blood tests revealed a substantial elevation in WBC to 27100/μl and C-reactive protein (CRP) to 29.3 mg/dL. Bilirubin and alkaline phosphatase (ALP) were normal. The patient received a diagnosis of cholecystitis and a right subdiaphragmatic abscess, leading to antibiotic therapy and percutaneous transhepatic biliary drainage because of the consideration of difficult operation (Figure 1).

**Figure 2:**
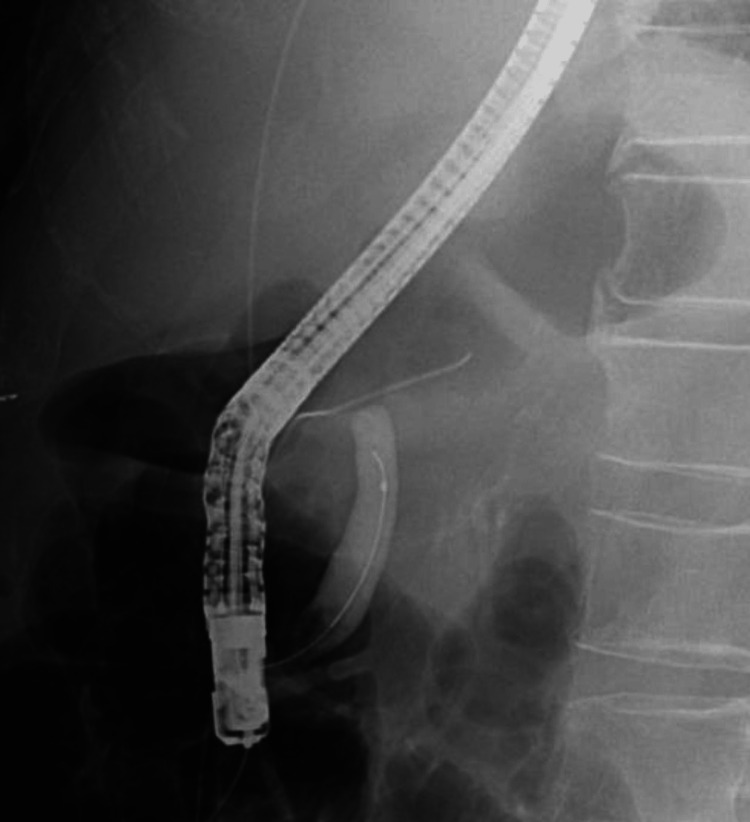
ERCP of the previous physician confirming BDI BDI: Bile duct injury; ERCP: Endoscopic retrograde cholangiopancreatography

Despite these interventions, there was no discernible improvement in the patient's condition. Consequently, it was determined that the patient would undergo cholecystectomy 16 days after commencing treatment. Due to the elevated preoperative inflammatory response and the presence of peribiliary abscess formation, there was a significant risk of severe adhesions, prompting the choice of laparotomy. Intraoperatively, as anticipated, a substantial degree of adhesion was observed surrounding the gallbladder. The adhesions of the omentum were disengaged, and scrutiny of the gallbladder unveiled necrosis and perforation at its fundus. The gallbladder and common bile duct were tightly adhered and required adhesiotomy. Inflammation in the proximity of the neck was likewise pronounced; however, the extraction of the gallbladder was executed by addressing a structure presumed to be the cystic duct. The duration of the procedure was one hour and 24 minutes, with a total blood loss of 100 milliliters. The morrow following the surgical procedure, his hematological examination revealed an augmented concentration of total and direct bilirubin, measuring 10.2 and 6.9 mg/dL, respectively, thereby culminating in a diagnostic inference of jaundice. Magnetic resonance cholangiopancreatography (MRCP) was conducted without yielding a definitive diagnosis. Consequently, endoscopic retrograde cholangiopancreatography (ERCP) was executed, leading to the ascertainment of a diagnosis encompassing BDI (Figure 2). The patient was in need of reoperation, but the absence of a biliary surgeon made it difficult to treat the patient at that hospital.

Six days after the initial surgery, the patient was referred to our hospital (HPB Center) for treatment. On admission, the physical examination revealed an abdominal wound under the right hypochondrium with a previously inserted drain into the foramen of Winslow. Very little effluent was observed from the drain. On medical examination revealed icterus in the sclera and integument, accompanied by mild tenderness primarily localized to the right hypochondrium. No spontaneous pain was observed. His body temperature registered at 36.7 °C, blood pressure measured 149/78 mmHg, and the pulse rate stood at 71 bpm. Blood test findings on admission to our hospital were as follows: white blood cell (WBC) 10100/μl, hemoglobin (Hb) 11.0 g/dL, platelet (PLT) 86.7×10^4^, prothrombin time (PT) 17.6 second, total protein (TP) 6.4g/dL, albumin 2.8 g/dL, total bilirubin 11.2mg/dL, direct bilirubin 8.8mg/dL, blood urea nitrogen (BUN) 15mg/dL, serum creatinine (SCre) 0.41mg/dL, sodium 128mmol/l, potassium 4.7 mmol/l, Cl 97mmol/l, aspartate aminotransferase (AST) 86 U/L, alanine aminotransferase (ALT) 42 U/L, ALP 681 U/L, C-reactive protein 2.13mg/dL. Approximately a month had elapsed when the patient presented at our medical institution, exhibiting a suboptimal general state, encompassing compromised nutritional status; consequently, the decision was reached to initiate the enhancement of his overall well-being as the initial step. A preoperative assessment was conducted via contrast-enhanced computed tomography (CT) scan, and percutaneous transhepatic cholangio-drainage (PTCD) was carried out to address jaundice.

**Figure 3:**
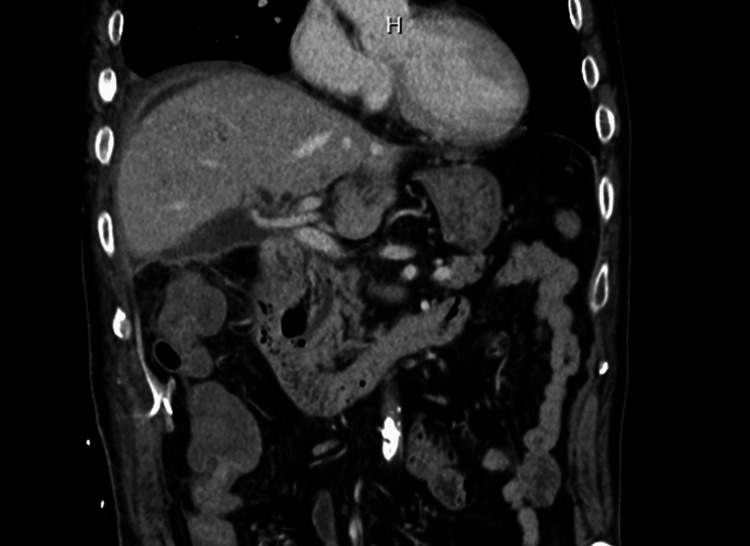
CT before reoperation showing disconnection of the common bile duct and dilation of the intrahepatic bile duct

The CT scan revealed that the connection between the common bile duct (CBD) and the common hepatic duct at the hilum of the liver was unclear, and there was dilation of the intrahepatic bile duct (Figure 3).

**Figure 4:**
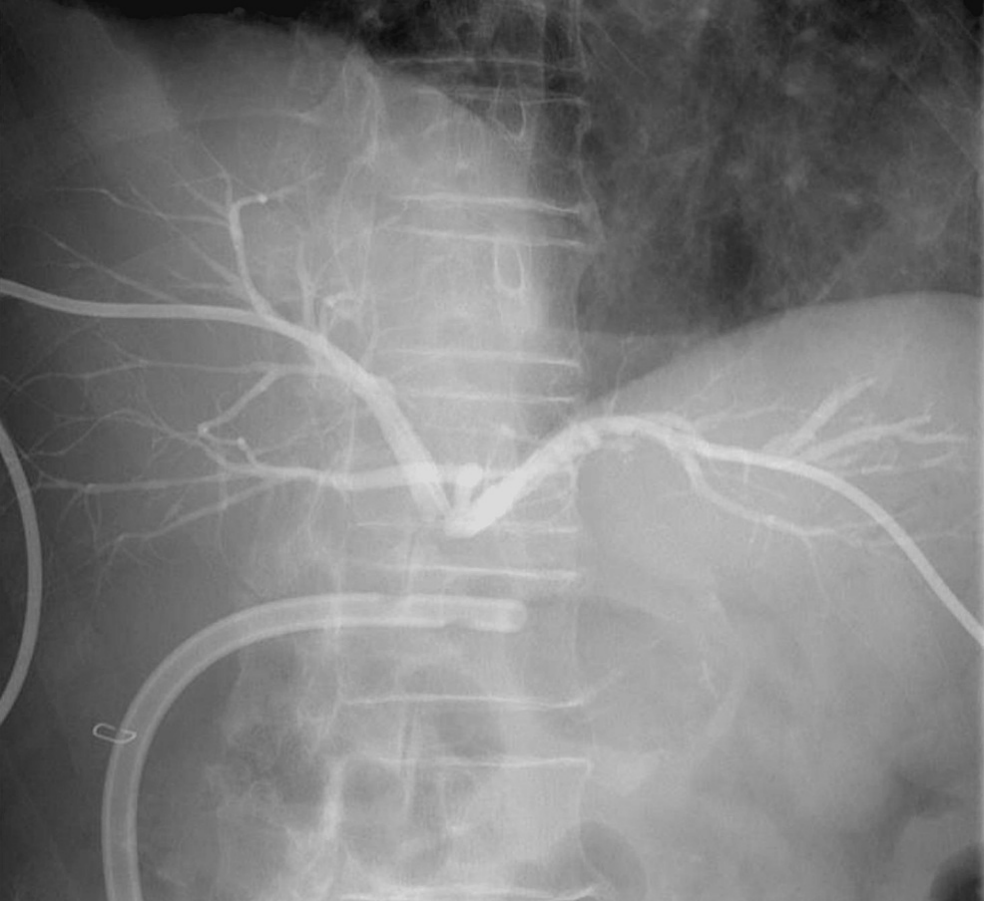
Cholangiography of the PTCD confirming discontinuity of the common bile duct in the hilar region (Strasberg bile duct injury E3) PTCD: percutaneous transhepatic cholangio-drainage

No vascular injury was identified on CT. Fluid retention was observed surrounding the hepatic hilum. Drainage tubes for the bile ducts were placed in the anterior segmental branch and B3. Cholangiography performed during the PTCD procedure showed a tear in the CBD (Figure 4).

Approximately a month subsequent to the patient's hospital admission, notable enhancements were noted in both nutritional status and a reduction in the manifestation of jaundice (Table 1).

**Table 1:** Preoperative blood test results

Tests	Values
WBC	6900/μl
Hemoglobin	10.6 g/dL
Platelet	47.1×10^4^
Albumin	3.8 g/dL
Total bilirubin	3 mg/dL
Direct bilirubin	0.7mg/dL
Blood urea nitrogen	12 mg/dL
Serum creatinine	0.54 mg/dL
Aspartate aminotransferase	41 U/L
alanine aminotransferase	46 U/L
C-reactive protein	0.08 mg/dL

Hepaticojejunostomy was deemed the appropriate course of action based on the aforementioned examination results. The preoperative assessments, including cardiac and pulmonary function tests, were uneventful.

Surgical procedure

**Figure 5:**
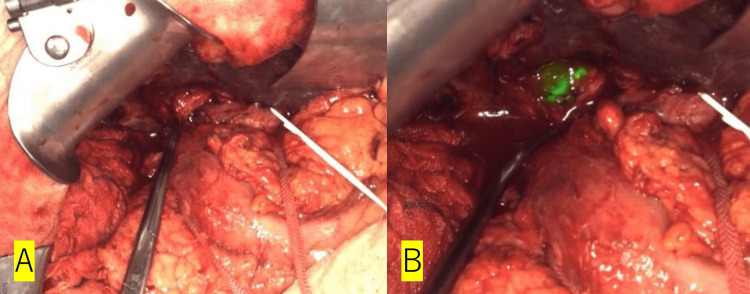
Confirmation of the bile duct stump by ICG (A) Before ICG injection; (B) After ICG injection. The exact location and extent of the CBD was identified. ICG: indocyanine green Camera system: LIGHTVISION (Shimadzu Corporation, Kyoto, Japan)

Approximately two months had passed since the initial surgery. The abdominal cavity was accessed via an additional upper midline incision at the site of the previous wound. Intra-abdominal adhesions were high degree due to the previous surgery. We performed adhesiotomy around the hilum of the liver and taped the hepatoduodenal mesentery, proper hepatic artery, and right hepatic artery. Nevertheless, we were unable to locate the stump of the CBD. Upon completion of the dissection at the hilum of the liver, noticeable scarring was observed in the location where the CBD should have been present. The full extent of the CBD remained unknown, and it was considered challenging to expose the CBD without risking damage to it. We decided to use ICG to accurately identify the location and extent of the CBD. ICG (2.5 mg/ml, 5 ml) was injected through the PTCD tube to confirm the localization of the CBD stump (Figure 5).

The stump was identified and the surrounding tissue was dissected. Following the hepaticojejunostomy, ICG was injected through the PTCD tube to evaluate the integrity of the anastomosis. Visible leakage was observed from the right lateral wall of the anastomosis after ICG injection, and additional sutures were placed to prevent further leakage. After additional sutures, ICG was injected again, but no leak was observed. Two drains, one anterior and one posterior to the anastomosis, were inserted. The duration of the procedure was five hours and six minutes, with a total blood loss of 120 milliliters.

Postoperative course

**Figure 6:**
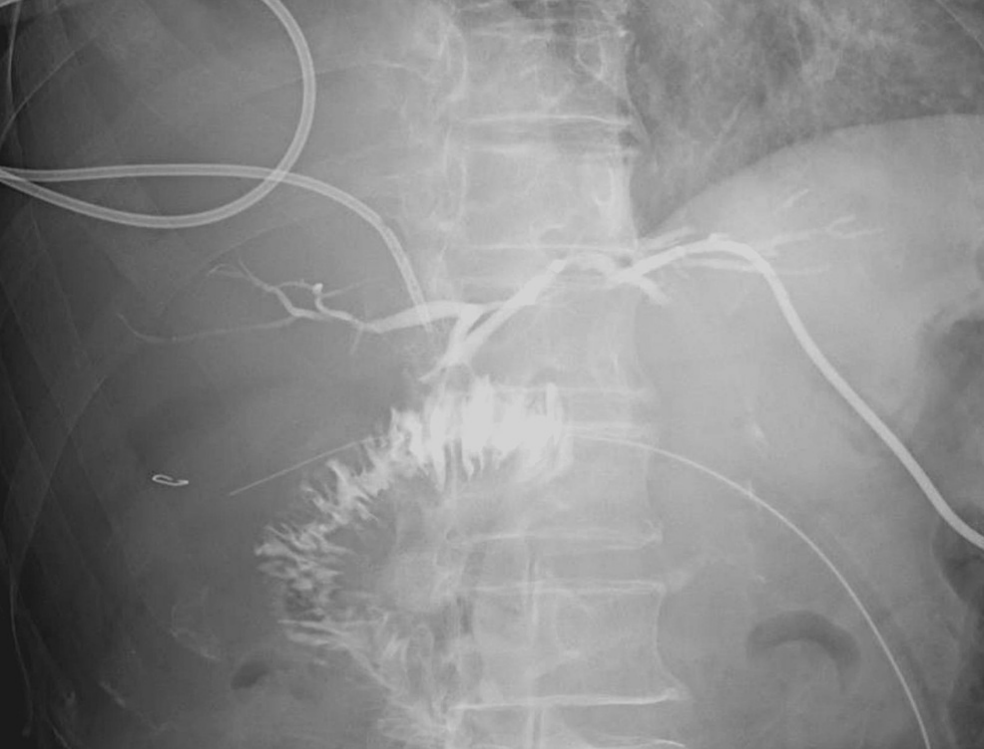
Postoperative cholangiography showing the absence of bile leakage

On the seventh day following the surgical procedure, cholangiography was executed with the utilization of a PTCD tube, wherein the absence of any suture malfunction at the anastomosis was verified (Figure 6).

No bile leakage was noted after clamping the PTCD tube, which prompted the drain removal on the ninth day after the operation. The PTCD tube was extracted on the 12th day, and the patient was released on the 13th day postoperatively. Two years have passed since the surgery and no major problems have been observed.

## Discussion

Bile duct imaging in cholecystectomy was first reported in 1937 by Mirizzi [5]. While contrast agents were conventionally used for radiographic imaging, more recent methods commonly employ biliary contrast with ICG [6]. ICG has the advantage of confirming the bile duct's location in real time without radiation exposure. As previously stated, ICG has recently been utilized for the prevention of BDI, and we have employed it in this case.

We believe that using ICG post BDI provides two main advantages. The first advantage is that it significantly enhances anatomical comprehension. In cases where a significant duration has passed since the occurrence of BDI, it is conceivable that adhesions around the bile duct may have solidified. In our case, bile duct location was challenging to identify without ICG. Some facilities do not have a biliary surgeon on staff, and situations requiring two-stage surgery may arise. We consider ICG an effective tool in such cases. Secondly, ICG can be used to detect bile leaks intraoperatively. In our case, ICG leakage from the bile duct jejunal anastomosis was identified during the operation. Therefore, we added sutures for the anastomosis and no bile leakage was observed postoperatively. Since biliary leakage is associated with postoperative prognosis and prolonged hospital stay, we believe that the method of using ICG is very useful from this aspect as well [7].

In the current case, a PTCD was inserted, therefore we utilized it. However, In some cases when a PTCD is not inserted, ICG may be administered transvenously. A study conducted a comparative analysis in which ICG was injected through direct puncture of the gallbladder and another where it was administered via the transvenous pathway. The group injected directly into the gallbladder showed a better contrast between the liver and the bile duct [8]. In situations involving the insertion of a PTCD or analogous device, as in our case, it may prove advantageous to consider its utilization. Upon the intrabiliary administration of ICG through the PTCD, the prompt identification of the bile duct stump was achieved in our case. On the other hand, it has been reported that ICG administered transvenously is recommended approximately one hour before imaging, and if ICG is used transvenously after the onset of BDI, we have to also take that time into account [9].

## Conclusions

Preventing BDI takes precedence as the primary objective, yet ensuring suitable treatment following the occurrence of BDI is crucial for the patient's quality of life. Particularly when a substantial duration has elapsed since the initiation of BDI, the presence of adhesions around the bile ducts can potentially complicate the process of identifying the bile duct's precise location. To ensure safe bile duct repair, ICG is one option that should be kept in mind when performing biliary surgery.
